
JOURNAL INFORMATION
==============================
NLM Title Abbreviation: South Med Rec
NLM Journal ID: 0027317

The Southern Medical Record

Publisher: Biomedical Journal Digitization Project

ARTICLE INFORMATION
==============================
PMCID: PMC9093577
Article version: 1
Subjects: Book Notices

An Index of Disease and Their Treatment

Print publication date: 1877 Dec 20
Volume: 7
Issue: 12
First page: 340
Last page: 340
License: This work is free of known copyright restrictions. For more information, please see PMC Back Issue Digitization.
License URL: https://creativecommons.org/publicdomain/mark/1.0/

==============================

An Index of Diseases and Their Treatment. By Thomas Hawks Tanner, M. D., F. R. S. Second Edition. Revised by W. H. Broadbent, M. D., Fellow of the Royal College of Physicians, Physician to the London Fever Hospital, etc., etc. One volume, octavo, 432 pages, price $3. Lindsay & Blakeston, Philadelphia.

The above volume is very practical and exceedingly useful to those whose time and circumstances will not permit them the use of an extensive library, the index, or alphabetical method, embodying a large amount and variety of information in a compact and very accessible form, by which the practitioner may promptly post himself in the definition of diseases, the origin and derivation of names, and the latest principles of treatment. To illustrate, we copy the following as a sample of the method adopted:

"*Pemphigus*, from *Pemphix;* (Greek) a bubble or blister. Synon. *Febris Bullosa; Bladdery Fever; Waterblebs*—A non contagious skin disease, characterized by large, round or oval vesicles, or bullae (*bulla*, a bubble of water), two or three inches in diameter, which appear on one or more regions, each bleb filled with alkaline serum, which soon loses its trans parency, becoming acid and puriform. Slight fever, etc., very liable to relapse.

*Pompholix* (*Pomphos*, a blister,) is merely a variety of *pemphigas*.

*Treatment.*—Arsenic usually most effectual; phosphorus; ammonia and bark; nitro-hydrochloric acid; quinine and steel; cod-liver oil; effervescing; citrate of magnesia; arsenic, quinine and steel; chlorate of potash; iodide of potassium; vesicle to be punctured; cuticle not to be removed."

The volume contains, in addition, an *Appendix of Formulæ*, and the various agents in the Materia Medica: *Uterine Therapeutics, Electro-Therapeutics, Climates for Invalids, Mineral Waters, etc.*

---

Dr. C. G. Polk again writes us, indignantly denying the charges made against him in the *Clinical Record and Hospital Gazette*, to which we referred in our last.

---

Receipted for 1877.—Drs. John Coston, A. H. Peek, R. L. Key, J. P. Rice, T. J. Fout, T. L. Darby, J. W. Huff, T. L. Quillan, '77 and '78.

### FOR SALE,

**Desirable Practice**

AND

**House containing four Rooms.**

30 miles from Little Rock, on L. R. & F. S. Railroad.

**Best opening in the State.**

$1,250 CASH.

Address **J. H. CALFEE, M.D.,**
Oct20-3*t* Conway, Ark.

**50 PER CENT TO AGENTS,** for the Illustrated Monthly, **UNION IN CHRIST,** **50** cents a year; also, large cash commission or valuabe premiums to canvassers for **THE LONDON CHRISTIAN HERALD**, an Illustrated Weekly, containing sermons of C. H. SPURGEON, DR. TALMADGE and D. L. MOODY, interesting articles, serial story, etc. Three Months, 75 cents, $2.50 a year, from U. S. Branch Office. Write now for sample copies and terms free, to H. A. KING, 17 Bible House, N. Y. City.
dec-12m

---

**THE NATIONAL AGRICULTURIST AND WORKING FARMER**, established 1847, a double quarto, 16 page, Illustrated Family Paper, devoted to Agriculture, Stock-Raising, Bee-Culture, &c. Try it! 6 momths for 55 cents, or with the ILLUSTRATED PRONOUNCING DICTIONARY, 320 pages, 250 engravings, cloth, sent with the paper a year, both postpaid, for only **$1.30**. Large commissions or valuable premiums to Agents. Address
WM. L. ALLISON,
dec-12m 128 Nassau St., N. Y.

---

### Physicians

—AND—

### LADY AGENTS

**$50 to $100 per week**

made easy by any

### Lady or Physician!

Most rapidly selling articles for married or single ladies' use.

**No Female Can do Without Them!**

**County and State Rights Free,**

**Lady AGENTS Wanted Everywhere!**

ADDRESS

**Bandanah Manf'g Co.,**
Oct20-12m NEW YORK.
